# Alternating Magnetic Fields Remove Biofilms but Damage Cells on Implant Models Also with Negligible Bulk Heating

**DOI:** 10.1021/acsami.5c12247

**Published:** 2025-08-26

**Authors:** Konstantin Nikolaus Beitl, Sandra Pérez-Jiménez, Guruprakash Subbiahdoss, Erik Reimhult

**Affiliations:** Institute of Colloid and Biointerface Science, Institute of Colloid and Biointerface Science, 27270 BOKU University, 1190 Vienna, Austria

**Keywords:** alternating magnetic field, cavitation, biofilms, magnetic heating, metallic implants, titanium, *Staphylococcus aureus*, osteoblasts

## Abstract

Implant-associated infections caused by bacterial biofilms remain a major clinical challenge, with high morbidity, often necessitating prolonged antibiotic therapy or implant revision surgery. To address the need for noninvasive alternatives, we investigated the use of alternating magnetic fields (AMFs) as a localized treatment modality for eradicating *Staphylococcus aureus* biofilms on titanium implant model surfaces. We demonstrate that AMF exposure effectively removes biofilms and kills bacteria at moderately elevated temperatures on the implant. Importantly, our results demonstrate that the antimicrobial efficacy of AMF treatment is primarily not due to heating. AMF vastly outperforms pure heating to the same temperatures for biofilm removal, despite inductive heating being the generally proposed mechanism for AMF antimicrobial action. Based on complementary imaging methods, we provide evidence that mechanical disruption, not a pure thermal effect, potentially driven by cavitation phenomena induced by transient, localized high temperature gradients, removes bacterial biofilms from titanium surfaces during AMF exposure. However, this mechanism also compromises the integrity of adjacent mammalian cells; confluent layers of SaOS-2 osteoblast-like cells exhibited actin cytoskeleton disintegration, membrane perforation, and a loss of viability even after brief AMF exposures. Our findings highlight a dual effect of AMF treatment: efficient biofilm removal is accompanied by collateral cytotoxicity, which requires further mechanistic research for clinically safe and effective AMF-based infection management strategies.

## Graphical abstract

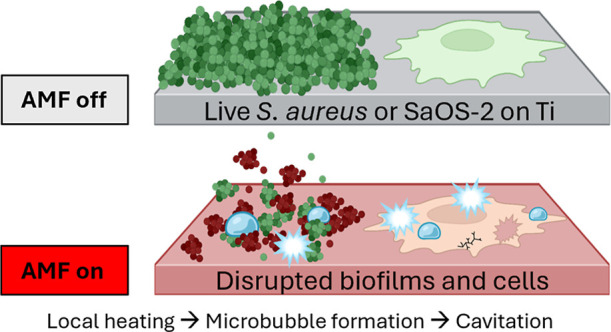

## 1 Introduction

Surgical implantation of metallic devices into the human body carries the risk of bacterial colonization on implant surfaces, which can lead to infection of surrounding tissues.^1^ This risk becomes especially pronounced when bacteria form biofilms that provide them with increased resistance to antibiotics and immune responses.^2^ Infections associated with biofilm formation can lead to long-term complications, which often require revision surgeries, exposing patients to additional risks of reinfection, and increased morbidity and mortality.^3,4^

Some common microorganisms associated with medical implant-associated infections (MIAI) are *Staphylococcus aureus*, *Staphylococcus epidermidis*, *Streptococcus pyogenes*, and *Pseudomonas aeruginosa*; several Candida species are also reported to cause MIAI, e.g., *Candida albicans*, *Candida glabrata*, *Candida parapsilosis*, and *Candida neoformans*.^5^ *S. aureus* is especially problematic as a multidrug-resistant pathogen.^6,7^ The dire consequences of MIAI stress the urgent need for effective and noninvasive biofilm eradication strategies to reduce healthcare-associated complications in patients dependent on surgical implantation of prosthetic devices.^8^

Various strategies have been explored to prevent or treat biofilms on implants without invasive surgical intervention. While prophylactic antibiotics have been traditionally used to mitigate infection risks, their overuse has significantly contributed to the global crisis of antimicrobial resistance.^9^ Modern approaches focus on the intrinsic prevention of biofilm formation by advanced material engineering.^10^ These include nonfouling coatings that prevent microbial adhesion through hydrophilic and zwitterionic polymers,^11,12^ and antimicrobial coatings with agents such as cationic polymers,^13^ enzymes,^14^ antimicrobial peptides,^15^ or engineered nanoparticles to actively inhibit microbial growth.^16–18^ Additional strategies to avoid the formation of bacterial biofilms on biomaterials include cell-adhesive coatings, which promote host cell integration while inhibiting bacterial colonization,^19^ and drug-releasing coatings, which deliver antimicrobial agents locally and in a controlled manner, minimizing systemic side effects.^20^ None of these approaches requiring some variant of surface coatings has been a general success, and biofilm-forming infections on implants are still too frequent occurrences.

Once bacterial biofilms have formed, extrinsic methods can be applied to treat or remove them in situ.^10^ Promising approaches for the noninvasive treatment of MIAI are physical techniques such as ultrasound, pulsed electromagnetic fields, and alternating magnetic fields (AMFs).^21–24^ Especially for metallic implants, AMF-based therapies offer unique advantages.^25^ AMFs induce heating through eddy currents generated by resistance in the metal, as well as through magnetic hysteresis losses in ferromagnetic materials.^26,27^ Apart from the reduction of bacterial biofilms, AMF-induced heating has already been demonstrated for a variety of medical applications, including cancer treatment and controlled drug delivery.^28–31^

When treating implant-associated infections with AMF, significant heating is required to eliminate bacteria and achieve sustained biofilm removal.^32,33^ This typically involves prolonged or high-temperature exposure, which risks damage to surrounding tissues.^34^ Thresholds like the cumulative equivalent minutes at 43 °C (CEM43) have been established to evaluate thermal tolerance.^35,36^ They assume that human tissue cells withstand heating better than biofilms.^37,38^ Nevertheless, excessive thermal exposure remains a key concern. Strategies such as applying AMFs in short, repeated intervals or combining AMF treatment with antibiotics have been proposed to mitigate thermal damage.^16,28,39^ Importantly, while heat is known to disrupt the rheological properties of biofilms and compromise their structural integrity, it may not fully account for the observed efficacy of AMF treatments in biofilm removal.^40,41^

While short-term moderate heating is unlikely to be effective on established biofilms, the fast local temperature increase from AMF at a metal surface can exceed the average temperature measured in the biofilm or surrounding tissue.^42^ The short-term high temperature could be more effective, but it is local. However, a rapid local increase in water temperature by the bursts of heat injection from AMF can lead to explosive microbubble growth and cavitation.^43^ So far, AMF-triggered cavitation has not been considered as a cause for biofilm disruption in the refs 41,42 . Fast microbubble formation and cavitation generate intense shear forces through phenomena such as microjet impingement and microstreaming.^44^ These forces can mechanically damage biofilms, facilitating their detachment and eradication.^45^ Using these effects, ultrasonic cavitation has been shown to remove bacterial biofilms from surfaces like dental implants.^46,47^ However, hydrodynamic cavitation can also harm mammalian cells and negatively affect cell viability.^48–51^ Hence, whether AMF removes biofilms through a purely thermal effect or through a thermally induced mechanical effect has major implications for its further development and the choice of parameters for determining safe use; e.g., the CEM43 guideline would no longer constitute a measure for safe application.

We decided to compare the response of *S. aureus* biofilms and a confluent layer of osteoblast-like (SaOS-2) cells on titanium substrates to alternating magnetic fields, focusing on tracing and explaining the strong effect demonstrated for AMF treatment, even at surprisingly short and moderate temperature increases. We grew biofilms on titanium surfaces and exposed them to the AMF for different durations. Biofilms were evaluated by using fluorescence microscopy and scanning electron microscopy (SEM). Additionally, we examined the effect on a confluent layer of SaOS-2 cells subjected to the same treatment. Our study provides new insights into the mechanisms making AMFs effective in removing biofilms from implant surfaces, implicating mechanisms beyond heating, with major implications for the use of AMF or noninvasive biofilm eradication on metallic implant materials.

## 2 Materials and Methods

### 2.1 Titanium Substrates

Titanium (Ti) sheets (Sigma-Aldrich, St. Louis, MI, USA) of 0.127 mm thickness were cut into 1 cm^2^ plates. The plates were cleaned by sonicating them in ethanol (≥99.8%; Sigma-Aldrich, St. Louis, MI, USA) and Milli-Q water for 5 min each, followed by UV/ozone treatment for 20 min. They were then autoclaved and kept under sterile conditions. Surface characterization was performed with water contact angle measurements and SEM (Figure S1).

### 2.2 Bacterial Cultivation and Biofilm Formation

The *S. aureus* strain ATCC 12598 (DSM 20372) used in this study was obtained from the DSMZ German Collection of Microorganisms and Cell Cultures GmbH (Braunschweig, Germany). They were first plated on tryptic soy broth (TSB; Sigma-Aldrich, St. Louis, MI, USA) agar plates from frozen stock solutions. A single colony was used for inoculation in 10 mL of TSB and allowed to grow overnight at 37 °C under aerobic conditions. After 16 h, the bacterial suspension was centrifuged at 3000 rpm for 5 min. The bacteria were resuspended in TSB + 1% glucose (Sigma-Aldrich, St. Louis, MI, USA), and the OD value was adjusted to 0.5 at 600 nm using a Hitachi U-2001 Spectrophotometer (Metrohm Inula GmbH, Vienna, Austria). For biofilm growth experiments, Ti plates were submerged in 1.5 mL of bacteria suspension in sterile 12-well plates (Avantor, Radnor, PA, USA) and incubated at 37 °C under aerobic conditions for 48 h.

### 2.3 SaOS-2 Cultivation

SaOS-2 osteosarcoma cells ACC 243 (DSMZ-German Collection of Microorganisms and Cell Culture GmbH, Braunschweig, Germany) were cultured as described in Ouni et al. using Dulbecco’s modified Eagle’s Medium (DMEM) with GlutaMAX, 10% fetal calf serum (FCS), and 25 mM HEPES (Sigma-Aldrich, St. Louis, MI, USA).^19^ Briefly, SaOS-2 cells were maintained in a T75 cell culture flask at 37 °C in a humidified 5% CO_2_ atmosphere. They were harvested at 95% confluency using TrypLE (Thermo Fisher Scientific, Waltham, MA, USA). The harvested cells were stained with a trypan blue solution (Thermo Fisher Scientific, Waltham, MA, USA) and counted using a Countess automated cell counter (Thermo Fisher Scientific, Waltham, MA, USA). Subsequently, 2 mL of DMEM-GlutaMAX complete medium containing 5 × 10^4^ cells/mL was seeded into each tissue culture well containing Ti plates. SaOS-2 cells were then incubated at 37 °C under a humidified 5% CO_2_ atmosphere for 48 h.

### 2.4 Alternating Magnetic Field Treatment

Ti plates supporting *S. aureus* biofilms or a confluent layer of SaOS-2 cells were transferred to glass vials containing 2 mL of 10 mM phosphate buffered saline (PBS; Sigma-Aldrich, St. Louis, MI, USA). The vials were then placed in a solenoid coil (dimensions: height × outer diameter × coil thickness × number of turns = 37 mm × 37 mm × 2 mm × 6) and exposed to an alternating magnetic field (AMF) of 60.9 A and 244 kHz generated by an Ambrell EASYHEAT LI (inTEST Corporation, Mount Laurel, NJ, USA). The product of the magnetic field and the frequency correlates strongly with the adverse effects from eddy currents in tissue, with a conservative upper limit of 5 × 10^9^ A m^–1^ s^–1^ for local hyperthermia treatment.^52,53^ Our exposure of ∼2.4 × 10^9^ A m^–1^ s^–1^ is below this limit. Samples were exposed to the AMF for different time durations, i.e., 10, 20, 30, 40, 50, or 60 s. Control samples were not treated with AMF. The temperature of bare Ti-plates was measured with an ETS-D5 electronic contact thermometer (IKA-Werke GmbH & CO. KG, Staufen, Germany) before and immediately after AMF exposure. After AMF exposure, the samples were removed from the vials for further investigation.

### 2.5 Bacteria Viability

The biofilms of AMF-treated and control samples were stained using the LIVE/DEAD *Bac*Light Bacterial Viability Kit (Thermo Fisher Scientific, Waltham, MA, USA) containing SYTO 9 and propidium iodide (PI). The staining solution was prepared by dissolving 3.34 mM SYTO 9, and 20 mM PI in PBS. The biofilms were incubated with 50 μL of the solution at room temperature and protected from light for 15 min. After rinsing with 1 mL of PBS, the Ti plates were inverted onto a glass coverslip, sealed with nail polish to prevent the biofilms from drying out, and imaged with a confocal laser scanning microscope (SP8; Leica Microsystems GmbH, Wetzlar, Germany). Images were taken at 40× magnification at randomly chosen points in the samples. Excitation: 488 nm; emission filter: 498–546 nm (SYTO 9) and 602–659 nm (PI). ImageJ (version 1.53t, U.S. National Institutes of Health, Bethesda, MD, USA) was used to quantify the surface areas of green and red signals in each image. Bacterial viability was calculated as the ratio of live (green minus red) to total green fluorescence area of the control, as SYTO9 stained all cells green. Percentages of live and dead bacteria (red) as well as removed bacteria were similarly determined as ratios relative to the total original biofilm area before treatment.

### 2.6 Quantification of Biofilm Surface Coverage on Titanium Substrate

Quantification of residual biofilm surface coverage after AMF treatment was performed with epifluorescence microscopy using a Nikon Eclipse TE2000-S (Nikon Europe B.V., Vienna, Austria) and scanning electron microscopy using an Apreo VS instrument (Thermo Fisher Scientific, Waltham, MA, USA). In the case of epifluorescence microscopy, *S. aureus* biofilms were stained with 0.1% crystal violet (CV; Sigma-Aldrich, St. Louis, MI, USA) for 10 min. After rinsing with PBS, the Ti plates were inverted onto a glass coverslip, sealed with nail polish, and imaged at 40× magnification at randomly chosen points in the samples. Excitation filter: 450–490 nm (SYTO 9) and 527–553 nm (PI); emission filter: 520 nm (SYTO 9) and 577–633 nm (PI). ImageJ was used to calculate the surface coverage of the residual biofilm after AMF treatment by taking the average of three biological replicates and five images per sample.

SEM micrographs were collected at 1000× magnification and randomly chosen points on the Ti disks (electron beam: 5–7 kV; 0.1–0.8 nA; detector: backscattered electrons). To this end, the samples were fixed after AMF exposure with 2.5% glutaraldehyde (Sigma-Aldrich, St. Louis, MI, USA) for 1 h at 4 °C. They were then rinsed with 10 mM PBS and gradually dehydrated in ethanol by carefully submerging the samples in increasingly concentrated ethanol solutions (25%, 50%, 70%, 95%, and 100%) for 3 min each. Subsequently, the samples were air-dried for 30 min and finally sputter-coated with 10 nm of gold using a Leica EM SCD500 sputter coater (Leica Microsystems GmbH, Wetzlar, Germany). ImageJ was used to quantify the surface coverage of biofilms by averaging biological triplicates and three images per sample.

### 2.7 Quantification of Residual Biofilm Biomass

Ti plates with CV-stained *S. aureus* biofilms (see above Section 2.6) were transferred to PBS-containing glass vials and sonicated for 5 min to detach all biological mass left on the plates after AMF exposure. The optical density of the resuspended biofilms was measured at 600 nm using a Hitachi U-2001 Spectrophotometer and was normalized against OD_600_ values of control samples, i.e., samples not treated with AMF.

### 2.8 Imaging SaOS-2 Cell Morphology

After AMF exposure, SaOS-2 cells were fixed with Roti Histofix (Carl Roth GmbH & Co. KG, Karlsruhe, Germany) for 10 min and were rinsed with PBS. For fluorescence microscopy, the cells were treated with 0.5% Triton X-100 (Sigma-Aldrich, St. Louis, MI, USA) in PBS (1 mL per well) for 3 min. After rinsing three times with PBS, the cells were stained for 30 min with 1% DAPI and 0.2% TRITC-phalloidin (Sigma-Aldrich, St. Louis, MI, USA) in PBS. After rinsing with PBS, the supporting Ti plates were inverted onto a glass coverslip and imaged using a Nikon Eclipse TE2000-S. Control samples were subjected to the same treatment, except they were not exposed to AMF. ImageJ was used for image processing.

For scanning electron microscopy, AMF-treated SaOS-2 cells or control samples (not exposed to AMF) were fixed, dried, and sputter-coated with gold, analogous to the procedure described above (Section 2.6).

### 2.9 SaOS-2 Viability and Recovery

SaOS-2 viability was assessed with epifluorescence microscopy. Control samples or AMF-treated cells were stained with ethidium homodimer-1 (EthD-1) and calcein AM (Thermo Fisher Scientific, Waltham, MA, USA) following a standard protocol: 50 μL of a staining solution containing 2 μM calcein AM and 4 μM EthD-1 were used to stain SaOS-2 cells for 30 min.^54^ For recovery, cells were further incubated in fresh cell culture medium for 48 h after AMF treatment as described above (Section 2.3) and then stained. After staining, samples were rinsed with PBS, the supporting Ti plates were inverted onto glass coverslips, and they were imaged using a Nikon Eclipse TE2000-S. ImageJ was used for image processing.

### 2.10 Statistics

Experiments were performed in three biological replicates. Images for quantification were taken in five randomly chosen areas in each sample. Mean data and standard errors are presented in the results section.

## 3 Results and Discussion

### 3.1 AMF Exposure Led to Gas Evolution and a Limited Linear Temperature Increase

To evaluate the effect of alternating magnetic fields on the temperature increase in titanium (Ti) plates, bare Ti plates (10 × 10 × 0.127 mm) were placed in glass vials containing PBS and positioned inside a solenoid. AMFs of 60.9 A and 244 kHz were applied for different durations of up to 60 s, and the temperatures of the Ti plates were measured. Figure 1A and B shows how AMFs were applied to heat titanium plates supporting *S. aureus* biofilms.

**Figure 1:**
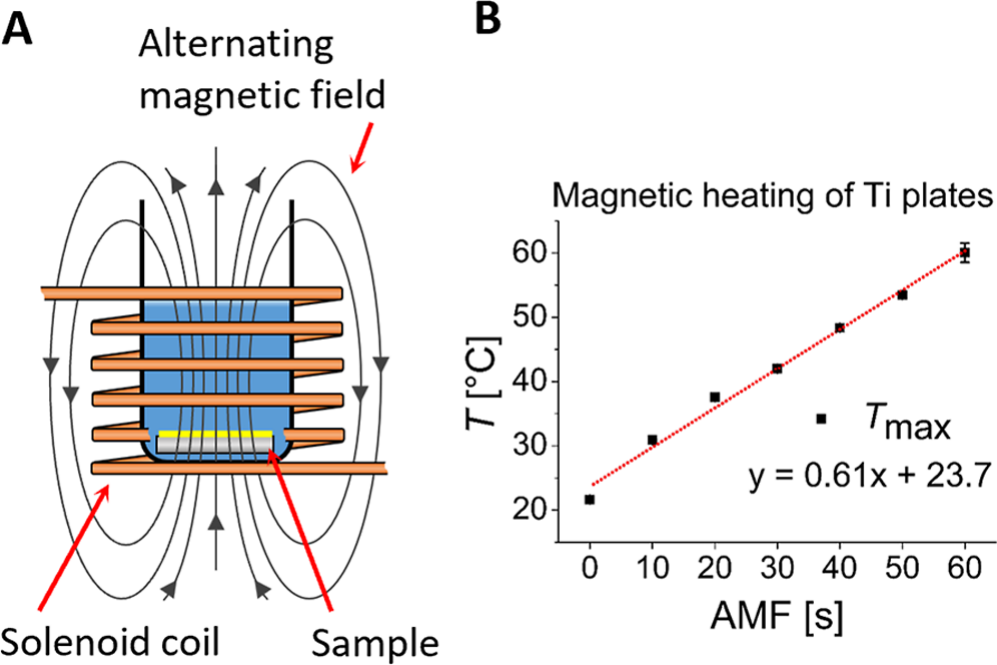
Heating of a titanium plate with an alternating magnetic field (AMF). (A) Schematic illustration of the experimental setup. (B) Titanium plate temperature measurements after exposure to an AMF for 10–60 s. Red dotted line: linear fit of maximum temperature measured (average of three measurements).

Upon exposure to AMF, the temperature of the bare Ti plates increased to approximately 31 °C from room temperature after 10 s. The temperature increased linearly with time at a rate of about 0.6 K/s and reached a maximum temperature of 60 °C after 60 s (Figure 1B). Local temperatures, however, could have been temporarily much higher during AMF heating than the average temperatures measured directly after heating; especially substrates with micron-scale roughness can show increased and localized heating.^42^ More importantly, the heat from the AMF is deposited in short bursts during the magnetic field cycle. The effect of localized delivery of high heat from rapidly heated surfaces has not been extensively investigated, but it has been demonstrated to lead to superheating, bubble nucleation, and even the formation of vapor blankets that collapse.^43^ However, acoustic effects from rapid localized heating by photothermal effects have been extensively investigated for decades, and three processes also relevant to localized AMF heating leading to microbubble formation, cavitation, and associated shear forces can be identified. First, rapid heating above 100 °C causes flash vaporization of the water. The collapse after the resulting compressive wave leads to rarefaction and cavitation.^55–57^ Second, biological media contain lots of gas, which similarly creates bubbles at much lower rapid temperature increases as their solubility decreases and the gas nucleates at higher temperatures, also leading to rapid microbubble formation, expansion, and shear forces. Third, strong local water volume expansion due to a rapid temperature increase compared to the surrounding water leads to an overpressure in the surrounding liquid.^58^ This compressional wave and trailing rarefaction zone can lead to cavitation, just as for flash vaporization. However, this so-called heat-shock cavitation can occur at much lower temperatures than 100 °C if the heating occurs rapidly.^59,60^

While microscopic investigation of bubble nucleation on the Ti substrate inside the magnetic heating coil was prohibitively difficult, we macroscopically observed effects consistent with transiently strong local heating and gas bubble formation. As observed in the video in the Supporting Information (Video S1), almost immediately after the application of the AMF, the reflection from the Ti surface changes, indicative of a rapid change in the refractive index near the surface, nucleating at numerous spots and spreading over the surface. As time progresses, thermally induced convection and the accumulation of larger gas bubbles visible to the eye are observed.

### 3.2 AMF Exposure Kills Most *S. aureus* in Biofilms on Titanium Plates

The effects of AMF exposure on the viability of *S. aureus* in biofilms were evaluated. For this, the remaining biofilms on Ti plates were stained with a bacterial viability kit (SYTO 9 and propidium iodide) after AMF exposure. As shown in Figure 2A,C, live bacteria stained with the membrane-permeable dye SYTO 9 fluoresce in green, while dead bacteria stained with PI emit red or yellow light (see also Figure S2).

**Figure 2:**
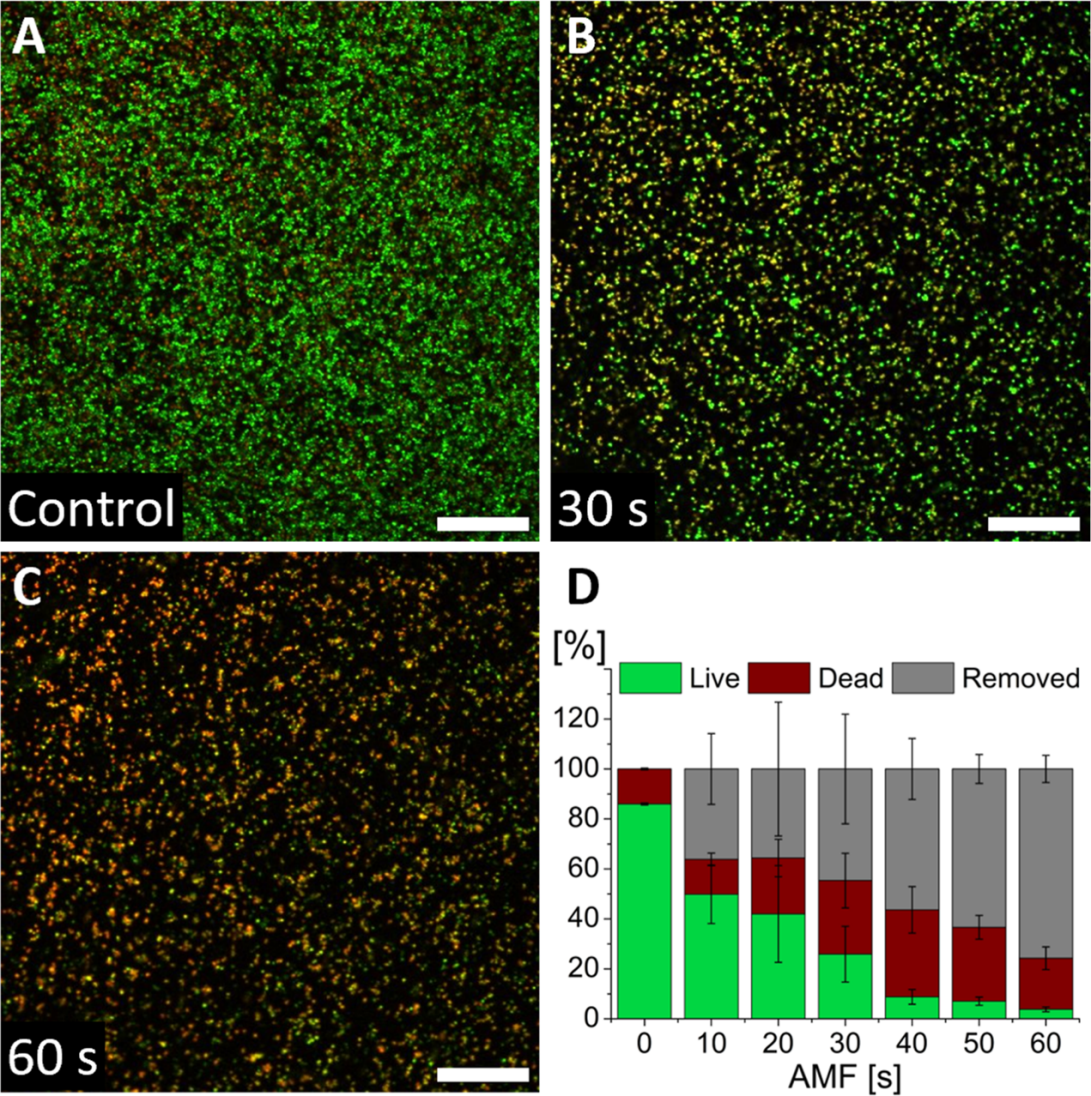
Representative confocal laser scanning microscopy images of *S. aureus* viability after AMF exposure (A) 0, (B) 30, and (C) 60 s. Green: live bacteria stained with SYTO 9; red/yellow: dead bacteria stained with propidium iodide. Images shown represent the average appearance of the samples. Scale bars: 50 μm. (D) Quantification of bacterial live (green) and dead (red) in the residual biofilm, and removed biofilm (gray), normalized to total bacteria surface coverage in the control.

Quantifying cell viability in terms of the relative area covered by live vs dead bacteria showed that increased AMF exposure resulted in increased bacterial killing (Figure 2D). The coverage in the control samples was 100%, and we calculated a *S. aureus* viability of approximately 86% ± 0.4%. Normalized to the total bacteria count in the control sample, the remaining viable fraction drops to 26 ± 11% after 30 s of AMF exposure; after 30 s of AMF exposure, the average temperature is 42 °C (see Figure 1B). This temperature roughly corresponds to the temperature in the CEM43 measure of tissue thermal load; i.e., at this temperature, our short exposures should not result in significant adverse tissue effects (see Figure S3). Within 10 s, about 36 ± 14% of the biofilm is removed with respect to control samples. After 30 s of AMF exposure, this value increases to 45 ± 22%. AMF exposure for 40 s, with an average Ti plate temperature of 48 °C, increased *S. aureus* killing compared to shorter AMF exposure times (see also Figure S2). Between 40 and 60 s of AMF exposure, corresponding to an increase to 54 °C average Ti disk temperature, the area covered by residual biofilm is further reduced from 56 ± 12% to 24 ± 5%. *S. aureus* viability dropped from 9 ± 3% to 4 ± 1% between 40 and 60 s (Figure 2D), normalized to the total number of bacteria on the control surface. However, the viable fraction of remaining bacteria in samples exposed for 40–60 s remained near constant at close to 20% (cf. Figure S2).

Several studies have shown that alternating magnetic fields can decrease bacteria’s viability, although experimental setups and AMF dosing differ.^28,39^ Wang et al., for instance, achieved a 3.29 log reduction of *S. aureus* colony forming units (CFU) by applying intermittent alternating magnetic fields (iAMF) to the biofilms.^28^ In this study, iAMF doses consisted of three exposures, each reaching maximum temperatures of about 65 °C within 3 s. The samples were allowed to cool for 5 min between exposures. In our study, we heat at a rate of approximately 0.6 K/s and do not exceed the tissue thermal dose (CEM43) up to 40 s of AMF exposure (CEM43 of 77.2 min reaching a maximum average temperature of about 48.4 °C; see Figure S3). The higher killing efficiency than that in our study likely results from more aggressive heating (about 9.3 K/s). Under the assumptions the authors make, both the tissue thermal dose (1 mm into the tissue) and the product of the magnetic field and the frequency are within clinically allowed ranges for local treatment.^34,35,50,52,61^ However, both the CEM43 and magnetic field exposures are significantly higher than in our study. Their much higher heat generation would also increase the bubble or cavitation-related phenomena.

### 3.3 AMF Exposure Significantly Removes *S. aureus* Biofilms from Titanium Plates

It is important to consider that if the biofilm remains and some bacteria survive, the residual biofilm comprises an ideal environment for reinfection, even at a low survival rate, and impedes the re-establishment of tissue. Hence, we quantified the removal of the biofilm, including its extracellular matrix, from the Ti plate surface. We used epifluorescence imaging of crystal violet (CV) stained biofilms (Figure 3A–C) and scanning electron microscopy (SEM, Figure 3D–F) to assess the reduction in surface coverage as well as morphological changes in the biofilm architecture due to AMF treatment as a function of time (see also Figures S4 and S5).

**Figure 3:**
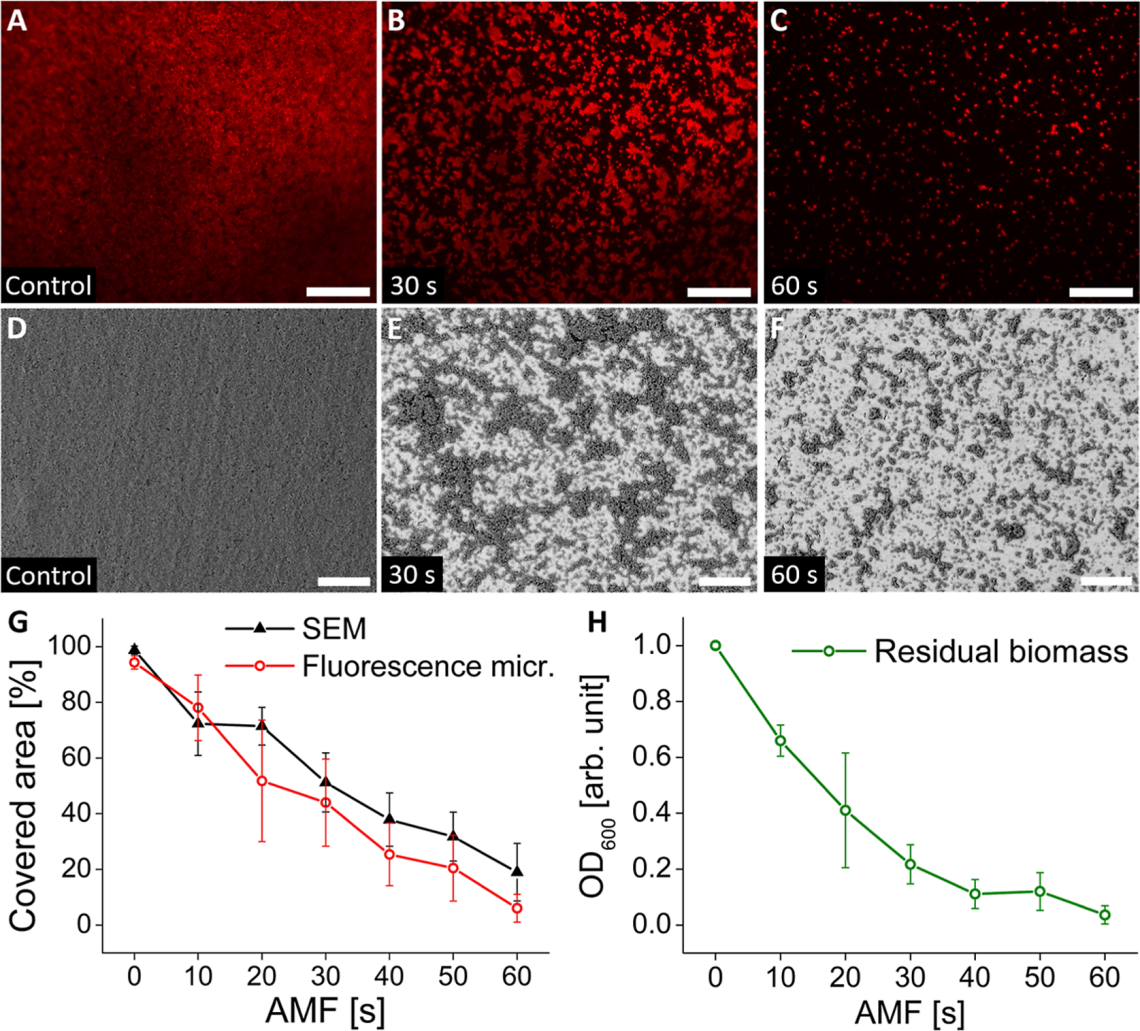
Representative images of *S. aureus* biofilms on Ti substrates after AMF exposure (0, 30, 60 s). (A–C) Epifluorescence microscopy of crystal violet-stained biofilms; (D–F) scanning electron microscopy; (G) quantification of biofilm surface coverage of samples imaged with SEM (black triangles) and epifluorescence microscopy (red circles); (H) OD_600_ measurements of the CV-labeled and redispersed residual biofilm mass normalized against control samples. Scale bars: 50 μm.

Figure 3 reveals locally progressing biofilm removal from the Ti substrate by AMF by all measures. Already after 10 s of AMF heating, gaps form in the biofilm that grow larger with longer treatment times and expose the underlying surface (Figures S4 and S5). After 30–50 s, these gaps have widened and merged, and only clusters of cells are left. After 60 s, the clusters remaining attached to the substrate are further reduced in size, approaching the size of single cells (Figure 3C,F).

As shown in Figure 3G, the biofilm surface coverage is approximately halved from close to 100% in the control after 30 s of AMF exposure (42 °C average surface temperature). Correspondingly, the normalized optical density of the CV-labeled dispersed residual biofilm mass at 600 nm (OD_600_) decreased by 78% to 0.22 ± 0.07 (Figure 3H). In samples imaged with epifluorescence microscopy, only 6 ± 5% of the titanium plate is covered by the remains of the biofilm after AMF exposure for 60 s (Figure 3C,G). The optical density of the CV-labeled and redispersed residual biofilm was reduced by 96% to 0.04 ± 0.03 after 60 s (Figure 3H). The surface area showing residual biofilm using SEM was generally higher than when imaged with epifluorescence microscopy, with 20 ± 10% of the surface still showing residual biofilm after 60 s AMF exposure (Figure 3G), but they agreed well with results for removed biofilm area obtained from the imaging of the bacterial cells with confocal microscopy (cf. Figures 2D and 3G). The trend of SEM showing a slower rate of removal of biofilm than fluorescence microscopy and the fastest rate of removal quantified by OD measurements of the released biomass is expected. SEM will image any material left on the surface without depth information, while OD will roughly quantify the volume of biofilm left. Cells adhere more strongly to the substrate than the surrounding matrix does, and epifluorescence microscopy measurements will show surface coverage if some stained material remains on the surface, even if most of the biofilm on top is lost. Hence, epifluorescence microscopy imaging of biofilm coverage should show a higher percentage of residual biofilm left than OD measurements.

The morphological appearance of the remaining biofilm structures shown in Figure 3 is similar to the respective images shown in Figure 2. Differences in how bacteria appear to be distributed over the surface between these figures can be largely explained by the different staining strategies. While CV primarily stains components of the extracellular matrix (ECM), it stains bacteria cells or bacteria clusters free of ECM less well than nucleic acid binding dyes used for bacteria viability staining do. Conversely, the DNA-binding dyes only weakly stain the eDNA in the ECM. The distribution of bacteria revealed by SEM imaging (Figure 3D–F) is strongly affected by how the samples are pretreated for imaging, i.e., fixing to the substrate, and the imaging technique itself, which requires drying the samples and exposing them to vacuum.

The amount of physical removal of biofilms using AMF heating is impressive. Other studies removing bacterial biofilms by temperature using heating baths have shown significantly lower efficiency at temperatures comparable to those in our and other AMF studies. For example, Richardson et al. used heat to treat biofilms formed on hemodialysis catheters by pumping hot water through the catheter lumen. After 2 h of exposing *S. aureus* biofilms to temperatures of 50 °C, about 50% of the cells were killed.^62^ Prasad et al. even reported that *S. aureus* biofilm reduction is ineffective or too time-intensive at temperatures below 60 °C.^38^ This suggests that the temperature increase alone is not driving the killing of bacteria, as AMF kills bacteria orders of magnitude faster and at average temperatures lower than those of global heating. It is particularly evident for biofilm removal, which does not occur at average temperatures close to body temperature, but we show a strong effect of AMF, progressing in discrete local areas.

As described in the introduction and the section “AMF exposure led to gas evolution and a limited linear temperature increase”, heating Ti-supported biofilms with an external AMF is fundamentally different from global heating. The heating is local, and the temperature on the surface can be much higher than the measured average temperatures or surrounding water and tissue temperatures, leading to microscale conditions potentially triggering various microbubble and cavitation-inducing phenomena, exerting destructive shear forces on any biological assemblies in the vicinity of the Ti–implant interface. Ultrasonic cavitation is known to efficiently remove biofilms, not only kill the bacteria, e.g., from the surface of dental implants.^47,63^ Our results show a combination of lower-than-expected viability and higher-than-expected local removal of the biofilm ECM over short intervals compared to studies on heat-induced biofilm removal as well as a rapid interfacial change, bubble formation, and strong heat convection. These observations fit the picture of AMF-heating-induced microbubble formation and cavitation, disrupting the biofilm and killing bacteria. The violent local mechanical forces unleashed by expanding and collapsing bubbles are a more reasonable explanation for removing chunks of ECM than dissolution within tens of seconds due to heating to temperatures close to body temperature.^46^

### 3.4 AMF Exposure Induced Structural Damage, Membrane Holes, and Cell Death in Confluent SaOS-2 Osteosarcoma Cell Layers on Titanium Plates

While tissue is relatively heat-tolerant and has shown robustness compared to biofilms,^37,38^ the localized nature of AMF heating, particularly AMF-heating-induced cavitation, could be very detrimental to cells at the implant interface and alter the balance between biofilm eradication and tissue safety. It would alter the assumption that AMF-induced biofilm removal can be evaluated as tissue-compatible based on only comparing it to the equivalent CEM43. Therefore, we investigated the effect of AMF heating on confluent layers of human osteoblast-like cells (SaOS-2) on Ti substrates with epifluorescence microscopy and SEM. The morphology of SaOS-2 cells changed with increasing AMF exposure times (Figure 4). Epifluorescence microscopy of SaOS-2 cells labeled with the nuclear stain DAPI and cytoskeleton stain phalloidin-TRITC revealed that the cells retain their structural integrity up to 20 s of AMF exposure (see Figure S6), and exhibit a morphology typical for healthy SaOS-2 cells.^64–66^ SaOS-2 cell nuclei maintained their round, compact shape throughout AMF treatment up to 60 s (blue, Figure 4A–C). The filamentous actins (F-actins) of the cytoskeleton (red, Figure 4A–C) lost their structure after AMF exposures longer than 20 s, i.e., average temperatures of 42–60 °C. The cells exhibit partially detached and distinct membrane holes, mostly exposing the underlying metal surface.

**Figure 4:**
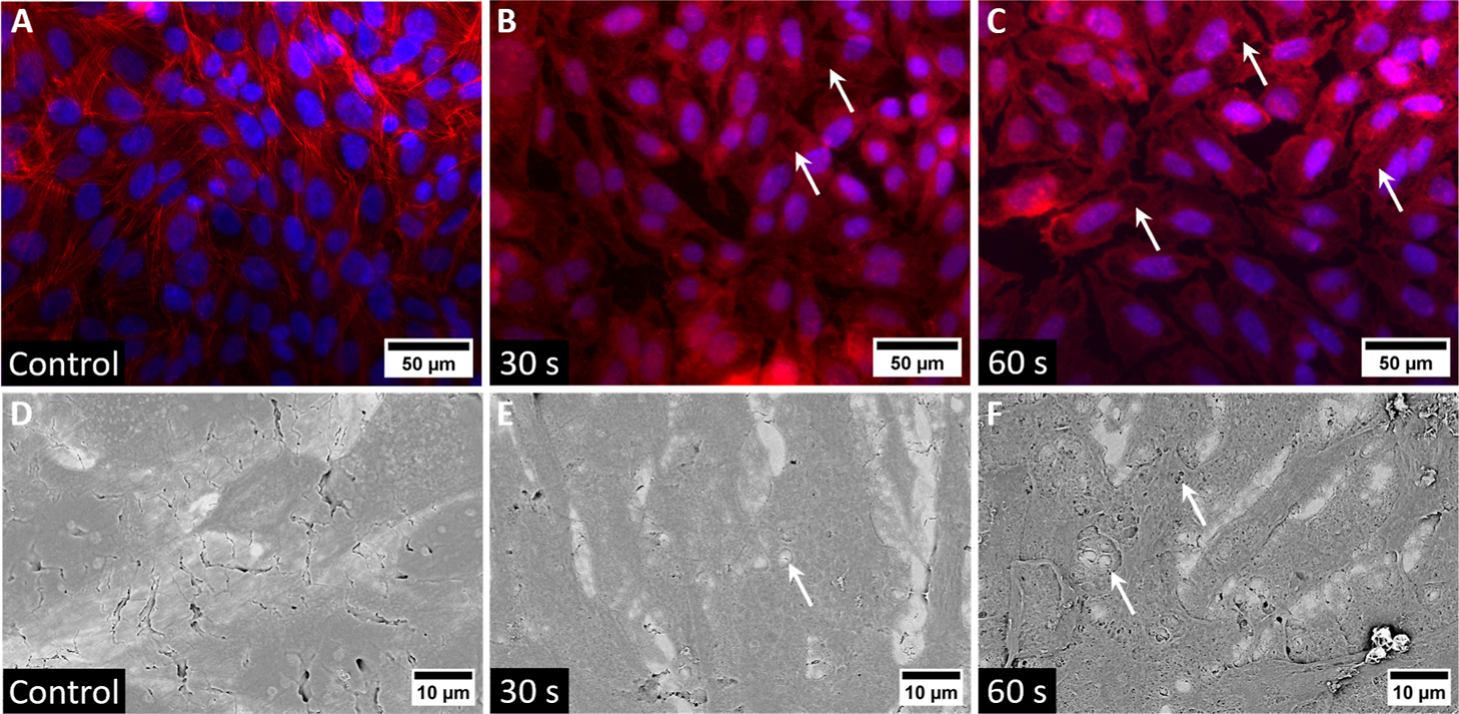
Representative images of SaOS-2 morphology after AMF exposure (0 s, 30 s, 60 s). (A–C) Epifluorescence microscopy. Blue: DAPI-stained cell nuclei; red: phalloidin-TRITC-stained actin filaments. (D–F) Scanning electron microscopy. White arrows indicate areas of cell damage, presumably due to cavitation, locally destroying the cell membrane and cytoskeleton.

Morphological changes like actin filament disintegration and membrane ruffling were observed by, e.g., Li et al. after heating osteoblasts for 10 min in water baths at 42 °C.^67^ Other groups reported changes in cytoskeletal structures after exposure to pulsed magnetic fields. Noriega-Luna et al. exposed human osteoblasts to a pulsed magnetic field of lower intensity than ours (0.65 mT at 4 Hz) for 45 min and observed a loss of actin filaments in the periphery of the cell membrane.^68^ Sadeghipour et al. similarly describe disruption and aggregation of F-actin after exposing human breast carcinoma cells to a 0.1 mT, 100 Hz pulsed electromagnetic field for 72 h.^69^ In another study, Ashdown et al. reported the formation of nanosized pores in the membrane of human lung cancer cells after treating them with a pulsed 20 mT field oscillating between 50 and 385 Hz.^70^ However, all of these studies involve low-frequency magnetic fields that do not cause elevated temperatures or cavitation, and the extent of morphological damage is both qualitatively different and lower.

On the opposite end, the safety aspects of AMF exposure of metal implants causing significant heating were first addressed by Chopra et al.^24^ They monitored the boiling of the liquid surrounding a metallic implant above 100 °C using a hydrophone in an animal model, but did not investigate or consider the damage caused before boiling occurred. Later, various groups were concerned with mitigating potential thermal damage by lowering exposure times through intermittent application of AMF, e.g., in combination with antibiotics, but not considering what type of damage occurred to cells and tissues.^28,71^

In contrast to previous studies, we observed additional types of cell damage occurring at temperatures close to body temperature. Large, round, dark areas were visible in the cell micrographs, showing complete local removal of the cytoskeleton and presumably the plasma membrane (some indicated by white arrows in Figure 4B,C). These severe structural cell damages are consistent with those expected from microbubble or cavitation-related phenomena.^48,51,72,73^ Strong mechanical forces are required to disassemble the cytoskeleton and the membrane to which it adheres within seconds. Additionally, the round shape and size of cell damage are consistent with the geometry expected from microbubble formation, cavitation, or jetting.

Scanning electron microscopy of confluent layers of SaOS-2 cells provides an ultrastructure-level view of the morphological changes. Images presented in Figure 4D–F highlight the damage caused by AMF heating. SEM reveals that SaOS-2 cells are damaged already after 10 s of AMF exposure, earlier than observed with epifluorescence microscopy (cf. Figures S6 and S7). Although the maximum average temperature reached only 31 °C within 10 s, large pores are already observed in cells, clearly showing injuries absent in the control samples. Upon longer AMF exposures, cells partially detach from the underlying substrate, and round-shaped holes are visible in the cells’ plasma membranes (indicated by white arrows in Figure 4). These defects become more common and more pronounced with longer AMF exposure times (Figure S6). After 40 s, reaching a maximum average temperature of about 48 °C, substantial damage led to the disintegration of the cells. Membrane pores are enlarged, and holes with diameters of several microns have been torn in the membrane, some exposing the underlying metal surface. These damages are found to an even stronger degree in samples that were exposed to AMF for 50–60 s (Figures 4 and S6).

The cell structural damage observed with SEM fully agrees with optical microscopy but provides further detail and mechanistic insight. Despite being recorded on the cell surfaces facing the bulk and not at the interface with the Ti substrate, it is evident that the structural damage is severe and localized on the submicron scale. The cell surface damage best fits the cavitation hypothesis, as cavitation will also occur at distances away from the surface in the wake of pressure waves. Similar cavitation-induced cell damage was reported by, e.g., Gevari et al.^73^ They studied the deformation of different cancer cell types under the effect of microscale cavitating flows and observed crater-like deformations of the cell body in the size range of the cavitation bubbles, detachment of filopodia, enlargement of membrane pores, membrane damage, and fragmentation of the cells. Hence, in summary, we propose that microbubble formation and cavitation caused by local heating explain the more than 1 order of magnitude earlier onset of morphological changes in our study than in comparable global heating studies on cells or bacteria. Unfortunately, this interpretation suggests that the higher heat resistance of host tissue cells compared to bacteria might not be leveraged for the most efficient forms of surface-based AMF-treatment.

### 3.5 AMF Exposure Induced Irreversible Membrane Damage to SaOS-2 Cells

Considering the drastic changes in cell morphology due to damage presumably caused by cavitation, we investigated whether the SaOS-2 cells were viable after AMF treatment and could recover by reintroducing the cells to culture for another 48 h. This would indicate a greater resilience of host cells than bacteria to AMF-induced damage. To this end, confluent layers of SaOS-2 cells on Ti plates were exposed to AMF for different time durations, reintroduced to culture for 48 h, and stained with the live–dead assay calcein AM and ethidium homodimer-1 (EthD-1) for epifluorescence microscopy imaging of viability (Figure 5).

**Figure 5:**
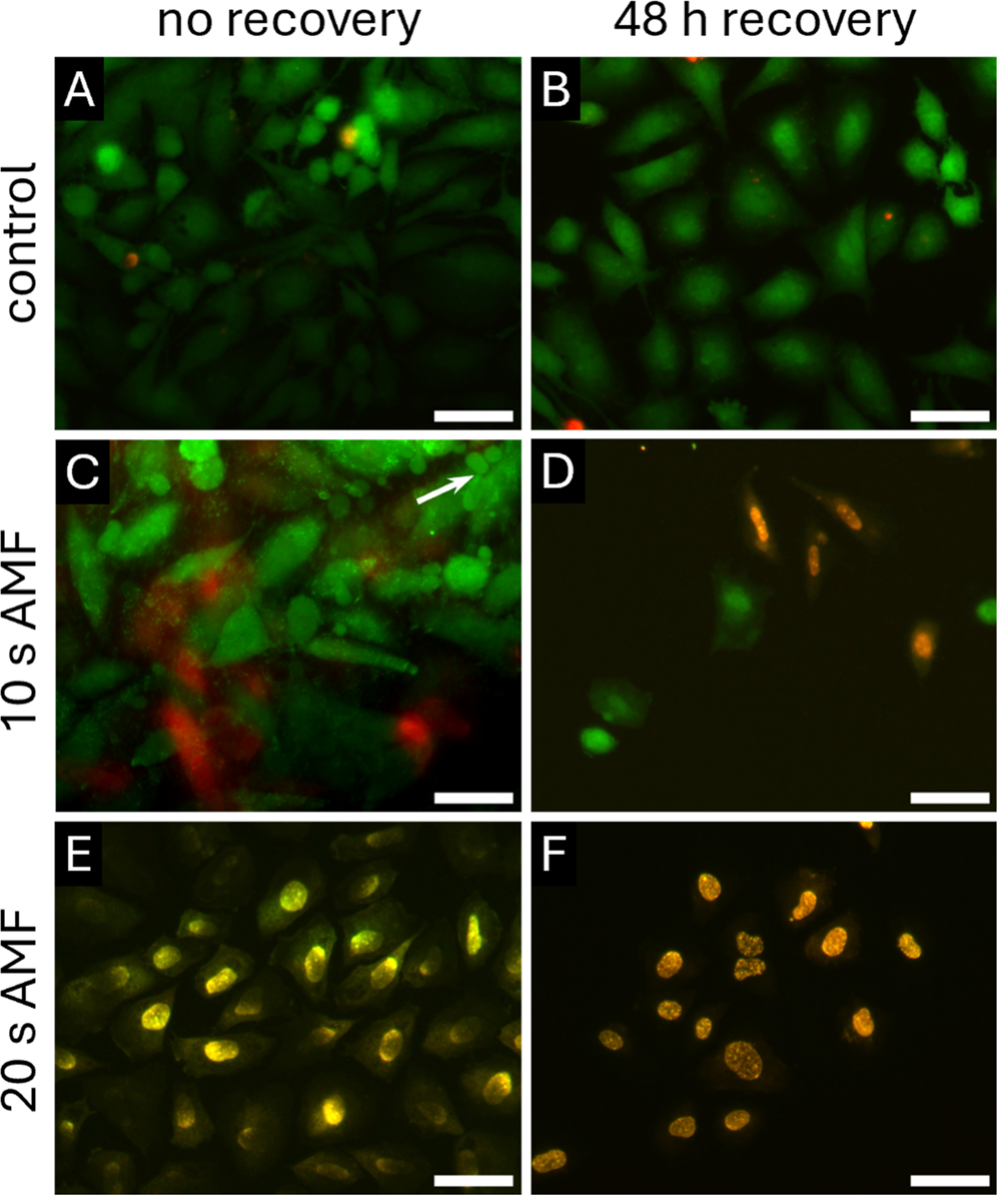
Representative epifluorescence microscopy images of SaOS-2 viability after AMF exposure and 48 h recovery incubation. (A) Untreated control, (B) control after further incubation for 48 h (‘recovery’), (C) osteoblasts exposed to AMF for 10 s (white arrow marks example for apoptotic bodies), (D) osteoblasts after 10 s AMF and 48 h recovery, (E) osteoblasts exposed to AMF for 20 s, and (F) osteoblasts after 20 s AMF and 48 h recovery. Green: Live cells stained with calcein AM; red/yellow: dead cells stained with ethidium homodimer-1 (EthD-1).

Control samples not exposed to AMF (Figure 5A,B) show viable cells and no difference in the cell number before and after the extended cultivation. Figure 5C shows that only a few SaOS-2 cells were recorded as dead (red or yellow) immediately after 10 s of AMF exposure. Indication of apoptotic cell death, such as plasma membrane blebbing and apoptotic bodies (white arrow), was observed for some cells still appearing green in a live stain.^48,63^ After reintroducing the treated cells to incubation for another 48 h after AMF exposure and performing live–dead staining and imaging, we determined that almost all SaOS-2 cells were dead, demonstrating that AMF exposure led to irreversible cellular damage, resulting in apoptosis. The cell number decreased drastically, and only a few cells remained attached to the Ti substrate (Figure 5D).

After AMF exposure for 20 s or more, all cells appear yellow after live–dead staining and imaging directly after the AMF treatment (Figures 5E and S8). It demonstrates the permeability of the cell membrane for the nucleic acid stain EthD-1 and presumed cell death. This result correlates with the extensive cell surface damage observed with SEM after 20 s of AMF exposure. No recovery of viable cells was observed after reintroducing the AMF-treated cells to culture for 48 h (Figures 5F and S8), and the cell number was further reduced. The cells showed no fluorescence signal from the cell lumen, indicating a sustained severe loss of membrane integrity.

Other studies have shown that osteoblasts can withstand higher temperatures than the maximum temperatures reached in our experimental design, depending on the duration of the exposure and tissue type.^37,74^ Cytotoxicity of thermal doses is commonly measured in Cumulative Equivalent Minutes at 43 °C (CEM43) to quantify heat resistance of different cell types.^61^ For bone tissue in rabbit models, irreversible bone resorption was reported after a CEM43 value of 16 min.^37,75^ Dolan et al. also report the resistance of osteoblast-like cells to mild heat shock.^76^ They subjected MC3T3-E1 cells to 45 °C for 30 s by exposing them to preheated culture medium and let them recover by returning to standard growth conditions (37 °C, 5% CO_2_). After 4 days of recovery, they reported no significant difference in the number of viable and apoptotic cells compared to nontreated control samples. Li et al. reported recovery of SaOS-2 cells exposed to 45 °C for 10 min within 12 h.^67^ The fact that SaOS-2 cannot recover from the experimental conditions chosen in our study, i.e., AMF exposure of 20 s reaching maximum average temperatures of 38 °C, further indicates that accompanying effects like microbubble formation and cavitation causing mechanical damage to the cell membranes and internal structure must be responsible for the high susceptibility of these cells to AMF treatment on Ti substrates.

## 4 Conclusion

Our findings demonstrate that AMF-induced biofilm disruption and adherent cell death are much more severe than expected from a mere short-term increase in temperature. The mechanism causing cell death and biofilm removal extends beyond the thermal effects. On the one hand, we demonstrated that AMF exposure greatly enhances bacterial killing and removes *S. aureus* biofilms on Ti surfaces compared to heating biofilms to similar temperatures. Clearly, AMF’s efficiency in quickly killing bacteria and removing biofilms depends on secondary effects, which, according to our results on biofilm morphology and bubble formation, are most consistent with mechanical damage from microbubble formation and cavitation. On the other hand, AMF caused irreversible structural damage and induced cell apoptosis in confluent layers of osteoblasts on Ti surfaces at even shorter exposures, under thermal conditions at which they should endure; the cells also showed membrane and cytoskeletal damage consistent with the mechanical stress expected from microbubble formation and cavitation. Comparing the impact of AMF on bacteria in biofilms and adherent cells, it is unclear if AMF could be tuned to remove biofilms without causing tissue cell death, at least in the adherent layer. Our findings highlight the need to carefully consider cavitation-induced damage to surrounding host tissues and not only thermal damage when applying AMF for implant-associated infections.

## Supplementary Material





## Notes

60 s AMF exposure of bare titanium (MP4)
Representative scanning electron microscope image of bare titanium surface, representative confocal laser scanning microscopy images of *S. aureus* viability after AMF exposure, calculation of CEM43 values for different AMF exposure durations, representative epifluorescence images of crystal-violet-stained *S. aureus* biofilms on Ti substrates after AMF exposure, representative scanning electron microscopy images of *S. aureus* biofilms on Ti substrates after AMF exposure, representative epifluorescence images of SaOS-2 morphology after AMF exposure, representative scanning electron microscopy images of SaOS-2 cell morphology after AMF exposure, and representative epifluorescence microscopy images of SaOS-2 viability after AMF treatment (PDF)

## Abbreviations

AMF: alternating magnetic field
CEM43: cumulative equivalent minutes at 43 °C
CFU: colony forming units
CV: crystal violet
DAPI: 4′,6-Diamidin-2-phenylindol
DMEM: Dulbecco’s modified eagle medium
ECM: extracellular matrix
EthD-1: ethidium homodimer-1
FCS: fetal calf serum
HEPES: 4-(2-hydroxyethyl)-1-piperazineethanesulfonic acid
MIAI: medical implant-associated infection
OD: optical density
PBS: phosphate-buffered saline
PI: propidium iodide
SEM: scanning electron microscopy
Ti: titanium
TRITC: tetramethylrhodamine isothiocyanate.
