# Supplementary material for: Alternating Magnetic Fields Remove Biofilms but Damage Cells on Implant Models Also with Negligible Bulk Heating
Source: ACS Appl Mater Interfaces. 2025 Aug 26;17(36):50432–42. doi: 10.1021/acsami.5c12247 (PMC12442004; doi:10.1021/acsami.5c12247)
Supplement: Supplementary file 2 [file am5c12247_si_002.pdf]

Supporting Information

Alternating magnetic fields remove biofilms but damage cells on implant models also with negligible bulk heating

*Konstantin Nikolaus Beitl, Sandra Pérez-Jiménez, Guruprakash Subbiahdoss, Erik Reimhult**

Institute of Colloid and Biointerface Science, BOKU University, 1190 Vienna, Austria

*Correspondence: erik.reimhult@boku.ac.at

This supporting information contains Figures S1 – S8 on pages S2 – S6.

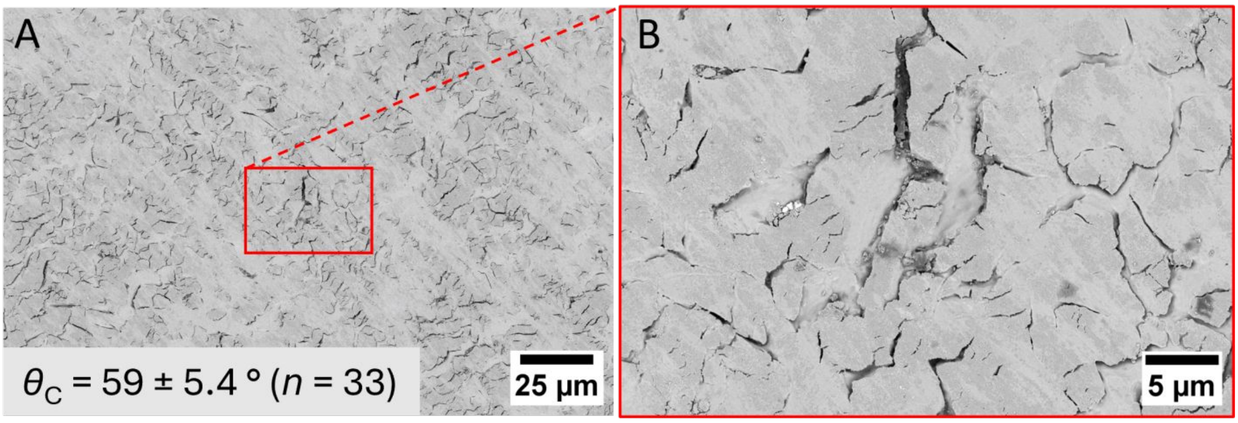

Figure S1. Representative scanning electron microscope image of bare titanium surface at 2000 × magnification (A) and 10.000 × magnification (B). Electron beam: 2 kV, 0.1 nA; detector: backscattered electrons. Water contact angle θ_C measured multiple times on 12 different samples as used ($n = 33$).

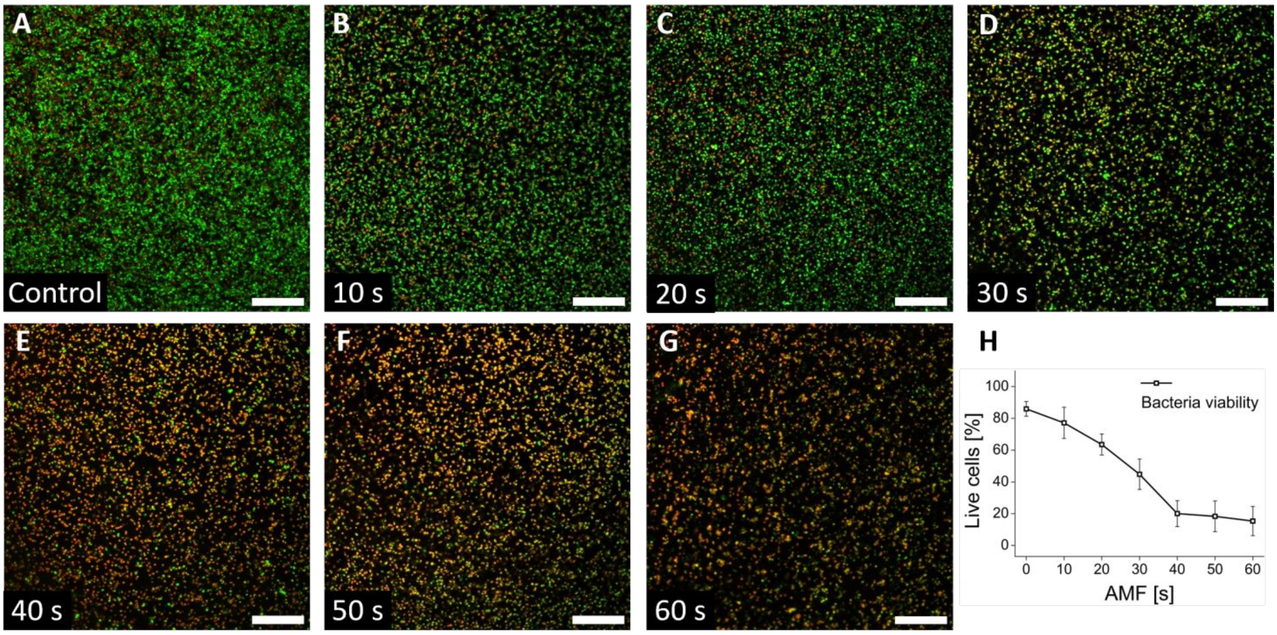

Figure S2. Representative confocal laser scanning microscopy images of *S. aureus* viability after 0 – 60 s AMF exposure (A) – (G). Green: live bacteria stained with SYTO 9; red/yellow: dead bacteria stained with propidium iodide. The selected images represent the average appearance of the samples. Scale bar: 50 μm. (H) Percentage of live bacteria in the remaining biofilms quantified in terms of surface coverage measured in five randomly chosen sample areas; three biological replicates each.

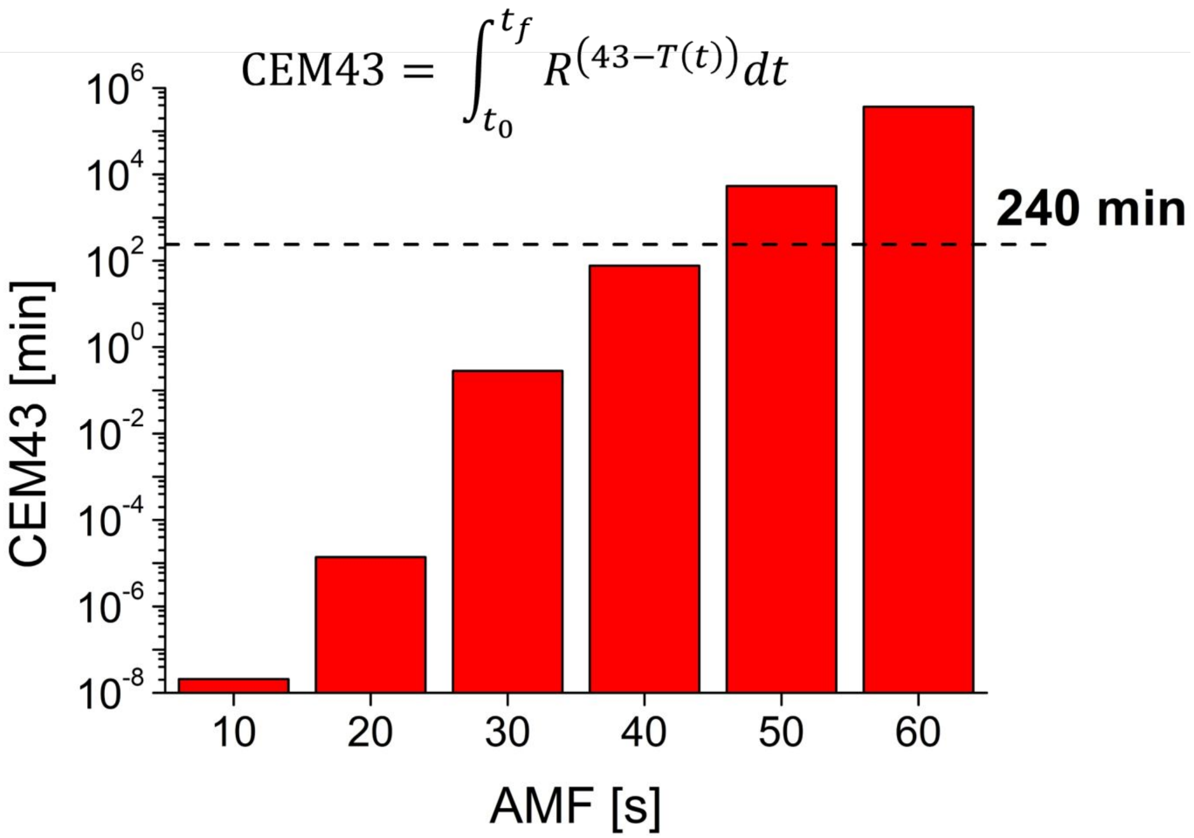

Figure S3. Calculation of CEM43 values for different AMF exposure durations, where t_f is the endpoint of AMF exposure, $R = 0.25$ for $T(t) < 43$ ˚C and 0.5 for $T(t) \geq 43$ ˚C, and $T(t)$ is the temperature. A cumulative dose of >240CEM43 minutes is considered to lead to significant thermal damage in many clinical applications and is indicated in the graph.

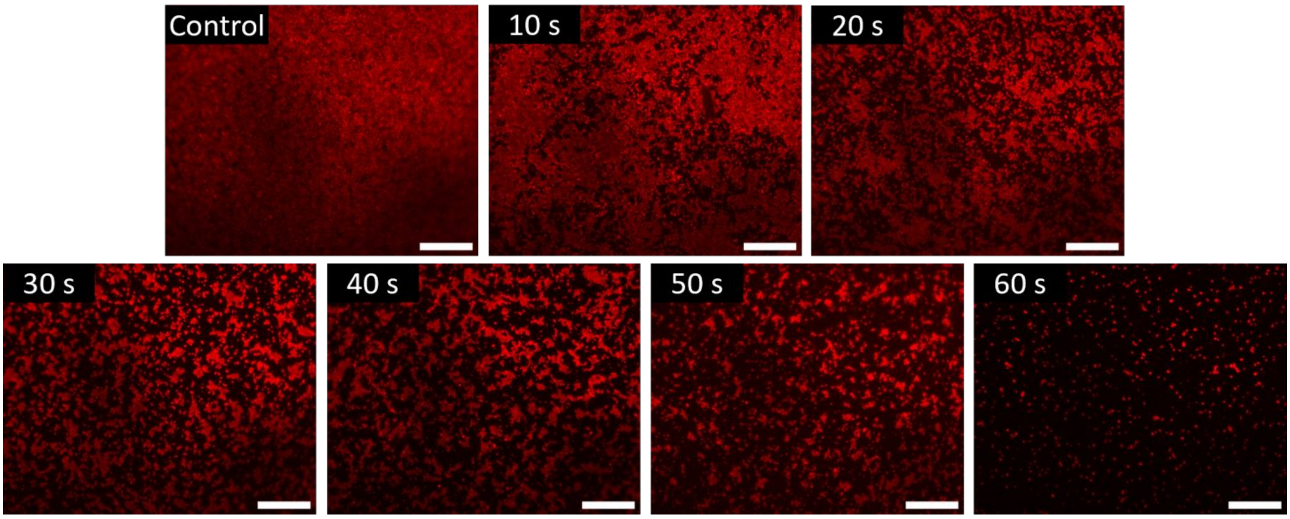

Figure S4. Representative epifluorescence images of crystal-violet-stained *S. aureus* biofilms on Ti substrates after AMF exposure (0 – 60 s). Scale bar: 50 µm.

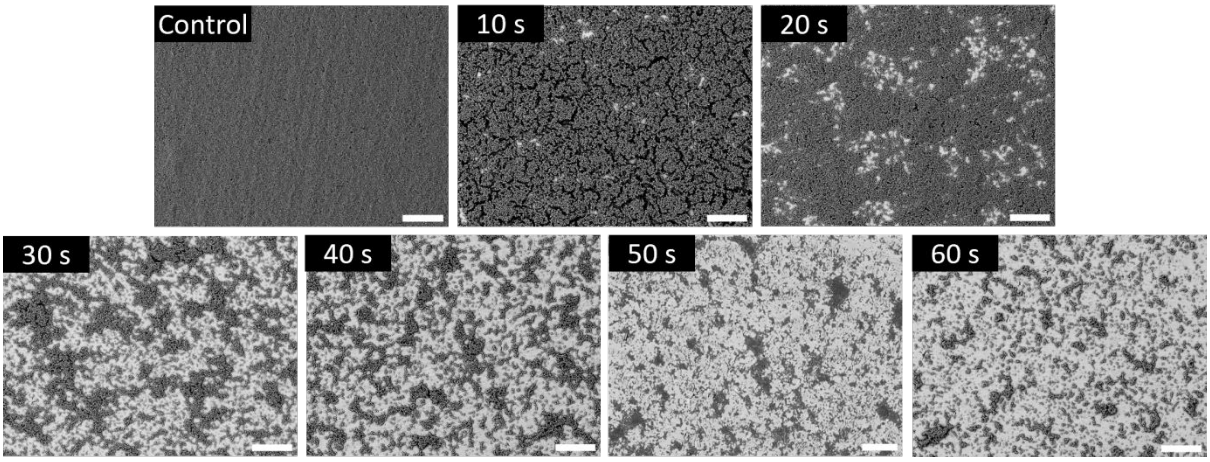

Figure S5. Representative scanning electron microscopy images of *S. aureus* biofilms on Ti substrates after AMF exposure (0 – 60 s). Scale bar: 50 µm.

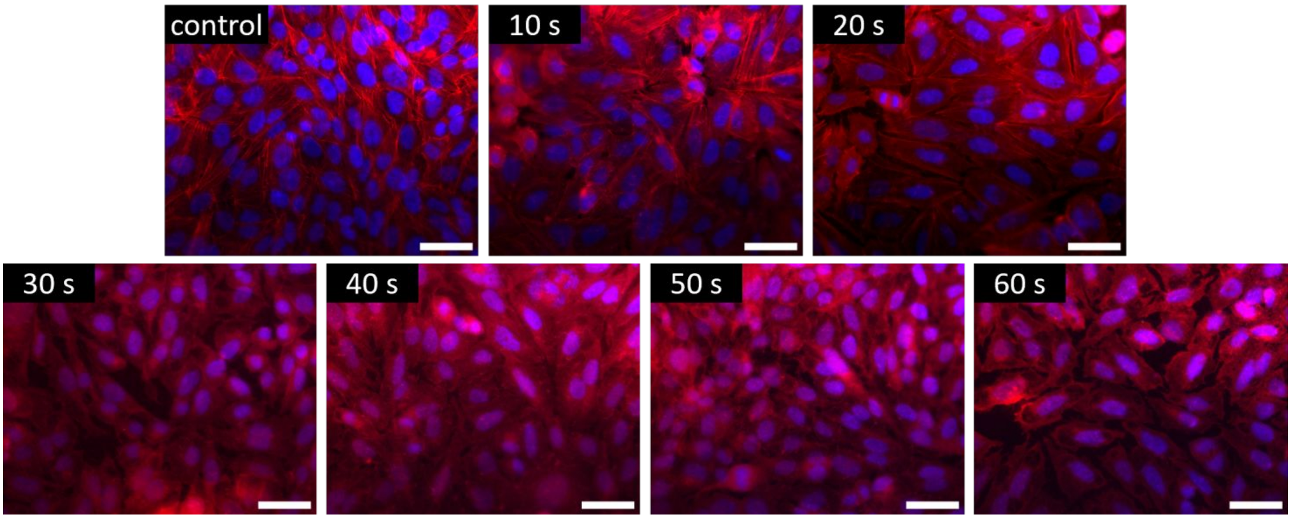

Figure S6. Representative epifluorescence images of SaOS-2 morphology after AMF exposure (0 – 60 s). Blue: 4′,6-diamidino-2-phenylindole (DAPI)-stained cell nuclei; red: phalloidin-TRITC-stained actin filaments. Scale bar: 50 µm.

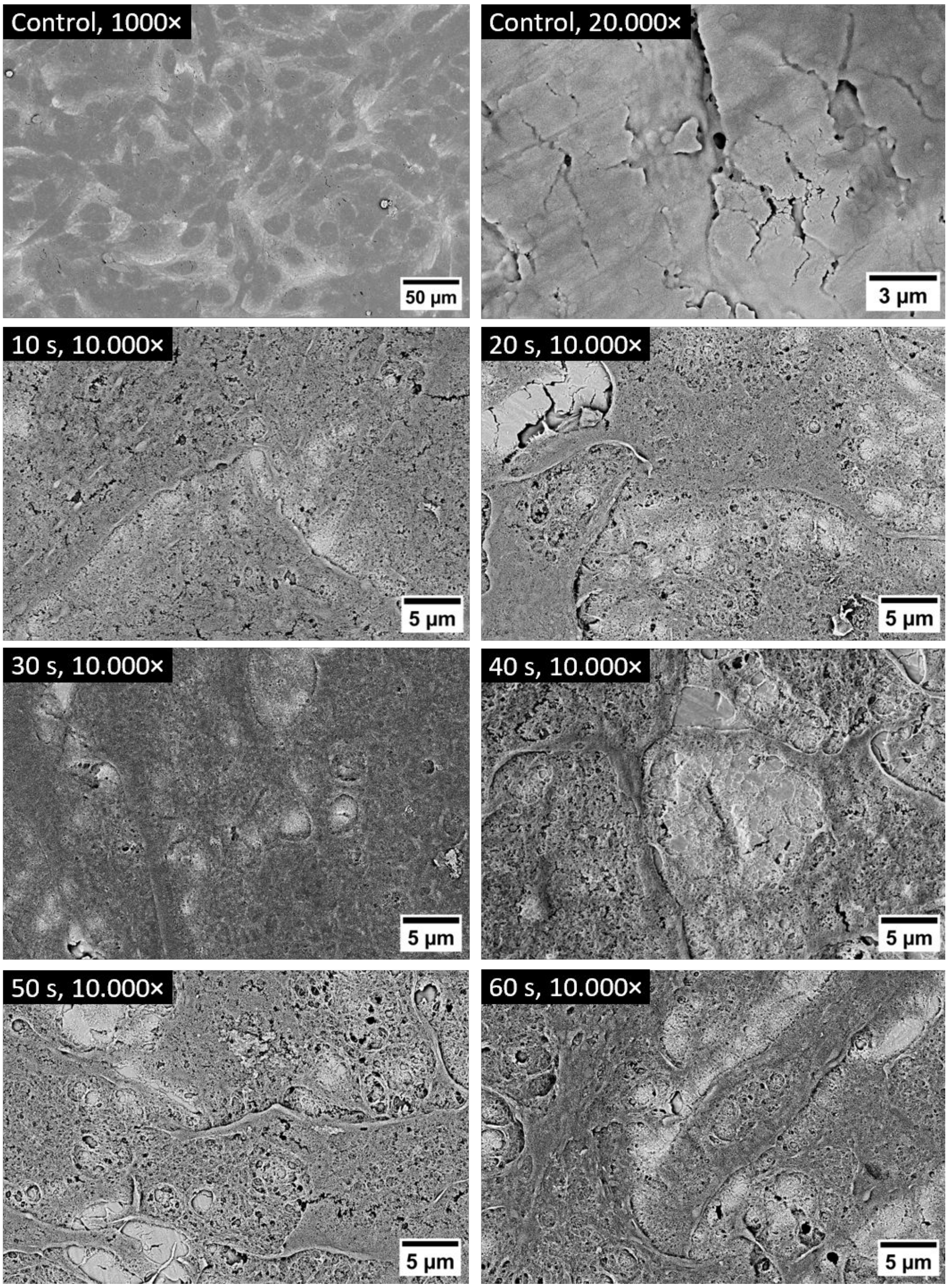

Figure S7. Representative scanning electron microscopy images of SaOS-2 cell morphology after AMF exposure (0 – 60 s) show cell detachment and membrane damage after AMF treatment due to cavitation.

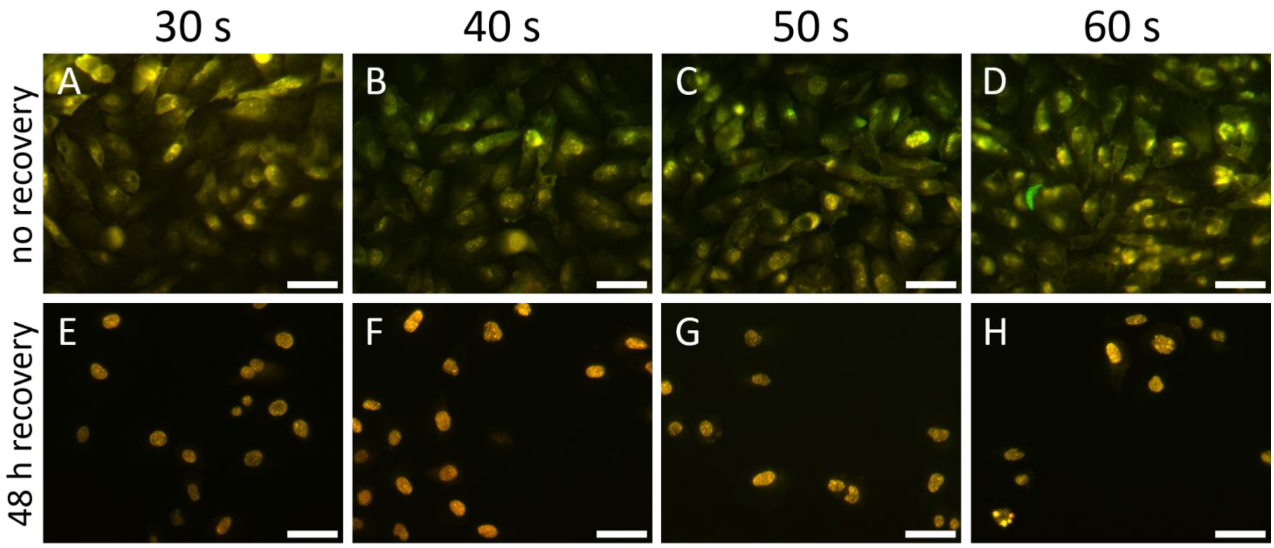

Figure S8. Representative epifluorescence microscopy images of SaOS-2 viability after AMF treatment (30 – 60 s). Top row (A – D): staining after AMF exposure; bottom row (E – H): staining after AMF and 48 h recovery. Green: Live cells stained with calcein AM; red/yellow: dead cells stained with ethidium homodimer-1 (EthD-1). Scale bar: 50 µm.

Video S1

A 60-second video showing the effect of alternating magnetic field exposure on bubble formation and convection for the Ti model substrate.
